# Shinrin-Yoku (Forest Bathing) and Nature Therapy: A State-of-the-Art Review

**DOI:** 10.3390/ijerph14080851

**Published:** 2017-07-28

**Authors:** Margaret M. Hansen, Reo Jones, Kirsten Tocchini

**Affiliations:** School of Nursing and Health Professions, University of San Francisco, 2130 Fulton Street, San Francisco, CA 94901, USA; rjjones2@usfca.edu (R.J.); kntocchini@usfca.edu (K.T.)

**Keywords:** Shinrin-Yoku, forest bathing, nature therapy, integrative medicine

## Abstract

**Background**: Current literature supports the comprehensive health benefits of exposure to nature and green environments on human systems. The aim of this state-of-the-art review is to elucidate empirical research conducted on the physiological and psychological effects of Shinrin-Yoku (or Forest Bathing) in transcontinental Japan and China. Furthermore, we aim to encourage healthcare professionals to conduct longitudinal research in Western cultures regarding the clinically therapeutic effects of Shinrin-Yoku and, for healthcare providers/students to consider practicing Shinrin-Yoku to decrease undue stress and potential burnout. **Methods**: A thorough review was conducted to identify research published with an initial open date range and then narrowing the collection to include papers published from 2007 to 2017. Electronic databases (PubMed, PubMed Central, CINAHL, PsycINFO and Scopus) and snowball references were used to cull papers that evaluated the use of Shinrin-Yoku for various populations in diverse settings. **Results**: From the 127 papers initially culled using the Boolean phrases: “Shinrin-yoku” AND/OR “forest bathing” AND/OR “nature therapy”, 64 studies met the inclusion criteria and were included in this summary review and then divided into “physiological,” “psychological,” “sensory metrics” and “frameworks” sub-groups. **Conclusions**: Human health benefits associated with the immersion in nature continue to be currently researched. Longitudinal research, conducted worldwide, is needed to produce new evidence of the relationships associated with Shinrin-Yoku and clinical therapeutic effects. Nature therapy as a health-promotion method and potential universal health model is implicated for the reduction of reported modern-day “stress-state” and “technostress.”.

## 1. Introduction

Research conducted in transcontinental Japan and China points to a plethora of positive health benefits for the human physiological and psychological systems associated with the practice of Shinrin-Yoku (SY), also known as Forest Bathing FB (FB) [1,2,3]. SY is a traditional Japanese practice of immersing oneself in nature by mindfully using all five senses. During the 1980s, SY surfaced in Japan as a pivotal part of preventive health care and healing in Japanese medicine [4]. The reported research findings associated with the healing components of SY specifically hones in on the therapeutic effects on: (1) the immune system function (increase in natural killer cells/cancer prevention); (2) cardiovascular system (hypertension/coronary artery disease); (3) the respiratory system (allergies and respiratory disease); (4) depression and anxiety (mood disorders and stress); (5) mental relaxation (Attention Deficit/Hyperactivity Disorder) and; (6) human feelings of “awe” (increase in gratitude and selflessness) [5]. Moreover, various contemporary hypotheses, such as: Kaplan’s Attention Restorative Hypothesis [6]; Ulrich’s Stress Reduction Hypothesis [7]; and Kellert and Wilson’s Biophilia Hypothesis [8] provide support and a lens for the practice of SY and other forms of nature engagement. 

**Figure 1:**
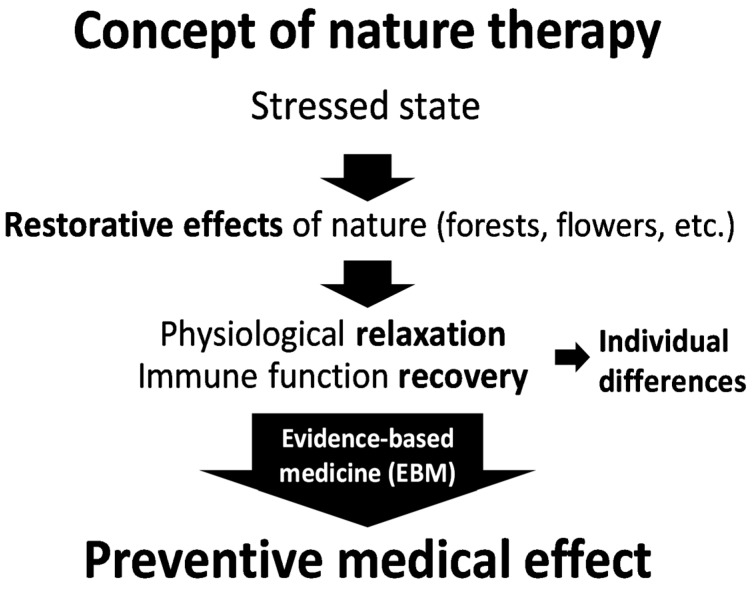
Concept of nature therapy [9]. Permission to publish from Yoshifumi Miyasaki.

Furthermore, SY may be considered a form of Nature Therapy (NT). Song, Ikei and Miyazaki’s present day model: Concept of Nature Therapy (CNT) [9] clearly defines NT as “a set of practices aimed at achieving ‘preventive medical effects’ through exposure to natural stimuli that render a state of physiological relaxation and boost the weakened immune functions to prevent diseases” [9]. The conceptual model of NT starts with a “stressed state” at the top and then points to the “restorative effects” of nature (forests, flowers, etc.) where there is a hypothesis of improvement in “physiological relaxation” and “immune function recovery” responses (individual differences noted). These responses to nature are then incorporated in the Evidence Based Medicine (EBM) model and is illustrated by an arrow leading to the “preventive medical effect.” This clear model supports Song, Ikei and Miyazaki’s [9] review of some medically proven outcomes. Kaplan and Kaplan [6] associated with exposure to naturally occurring stimuli (all 5 senses) that has a direct effect on increasing the parasympathetic nervous system and a heightened awareness that leads to a state of relaxation (Figure 1).

Individuals living and interacting in green spaces (GS) report being more energetic, in good overall health and, have more of a sense of meaningful purpose in life [10]. Current scientific findings are illuminating what humans intuitively know: nature has great benefits for the human brain and this is shown through increased happiness, health/well-being and cognition [5]. Historically speaking, Cyrus the Great intuitively built lush green gardens in the crowded urban capital of Persia 2500 years ago to increase human health and promote a sense of “calm” in a busy city. The 16th Century Swiss-German physician, Paracelsus, declared: “The art of healing comes from nature, not from the physician” [5]. These insights have lead SY researchers to investigate the modern health benefits of humans being exposed to nature or GS. 

Several studies explored the therapeutic benefits of SY in Asian countries [2,3,9,11]. Physiological and psychological differences between participants in a “forest therapy (FT)” program and a control were examined in the Seoul Metropolitan area with findings of a significant reduction in chronic widespread pain and depression [3]. Song and colleagues [9] demonstrated how male Japanese students who walked 15-min in an urban park during the autumn season had decreased stress and heart rates. By using several valid psychological tests, researchers demonstrated the positive effects of FT on individuals coping with chronic side effects of a cerebral vascular accident - specifically anxiety and depression [2]. At the Center for Environment, Health, and Field Sciences, Chiba University, Japan, researchers measured oxyhemoglobin levels in the pre-frontal cortexes of research participants while the participants observed three dracaena plants [11]. Results indicated a significant increase in participants’ oxyhemoglobin levels for urban, domestic and workplace foliage effects which directly demonstrates the health-promotion effects associated with indoor foliage plants on humans [11]. 

While exploring recent research about the health benefits associated with SY a dearth of scientific research conducted in Western populations was determined. Therefore, the increasing interest and the current published significant research findings surrounding the healing benefits related to SY, GS and the wilderness offers healthcare professionals an opportunity to delve deeper into this complementary modality for the prevention of disease and to assist with the potential healing of certain existing conditions in Western cultures. Revealing current research methods and subsequent research outcomes associated with SY practices may provide researchers, clinicians and students with an intervention that assists with preventative medicine and evidence-based practice (EBP). Therefore, the aim of this paper is to offer: (a) an in-depth inquiry of the current literature, (b) invite researchers residing in Western cultures to design and conduct empirical research regarding the therapeutic benefits associated with SY and, (c) to encourage healthcare providers/students to consider practicing SY to decrease undue stress and potential disconnection.

## 2. Materials and Methods

### Review Method

The terms of this comprehensive review were to emphasize the core elements of the research proposition. The initial literature search was conducted with the intention of identifying publications that offered significant historic relevance to the practice of SY, included various populations, sample sizes and geographic locales, utilized evidence-based practices, illustrated measurable physiological and psychological effect parameters, expounded upon practical frameworks and methodologies for the practice of SY, explicated unique measurable criteria for the application of SY and deduced limitations of previous research. 

#### Search Method

**Figure 2:**
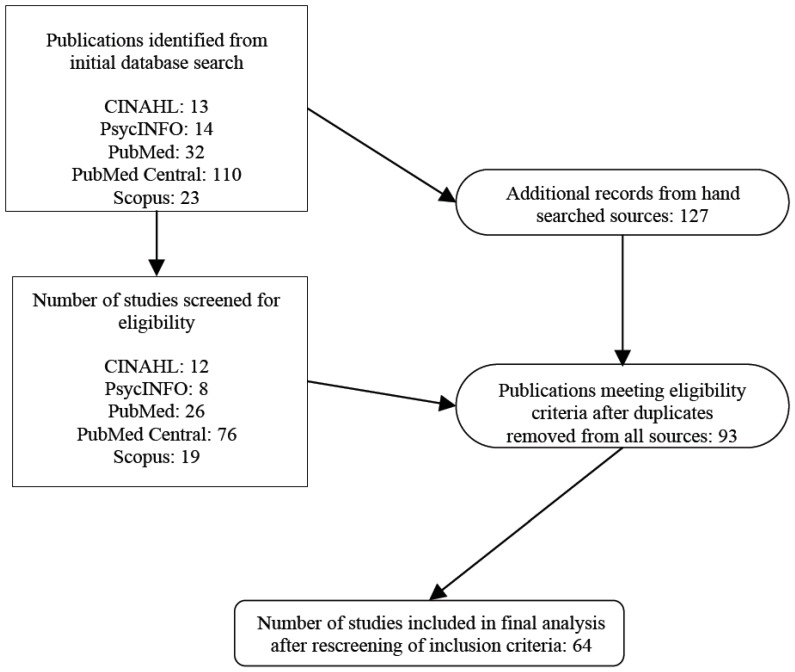
Literature search process.

The electronic databases searched included PubMed Central, PubMed, CINAHL, Scopus, and PsycINFO (Figure 2). Hand searched bibliographies and reference lists from seminal researchers of SY were also applied to the initial culling of publications. PubMed Central was searched to ensure the incorporation of relevant publications not indexed in PubMed. Keywords were used for each database and during snowball searches. All titles and abstracts were searched with the following terms: “shinrin-yoku,” “forest bathing,” and “nature therapy.” These searches were combined with the Boolean operators AND/OR. These terms were chosen from careful analyses of supporting literature. For example, the aforementioned terms “nature therapy”, “shinrin-yoku”, and “forest therapy” are used in conjunction with one another in the most recent scholarly literature review of NT in Japan [9].

To remain prescient, the reference range utilized in this review included literature published between the years 2007 and 2017. Therefore, the inclusion criteria allowed for publications that were available in English, dated from 2007 to 2017, incorporated transparent evidence based practices in reviews or trials, included robust quantitative and/or qualitative data, offered unique frameworks and theories, and explored current trends in research. Studies not meeting the tenets of this criteria, specifically those that pertained to physical exercise, fitness, landscape architecture, and laboratory, or animal studies were withdrawn from considerations.

## 3. Results

The findings of all relevant studies were synthesized (Table 1). The initial literature search revealed a series of topical themes apropos of the research aim. Articles were grouped into categories reflecting upon their most pertinent features. These categories include Background information, Frameworks, Physiological and Psychological effects, Sensory Metrics, and Limitations to findings. Previous Systematic Reviews and Literature Reviews were identified. Characteristics of publications specific to the themes of Physiological and Psychological Effects (PP), Sensory Metrics (SM), which is a subtopic of PP, and Frameworks (F) are delineated, an explicated within the key in Table 1. 

### 3.1. Physiological and Psychological (PP) Effects

Livni [12] published an editorial on the health benefits of SY and described the historic trends in biophysical and psychosocial research. While news of the beneficial elements of SY has been gathering momentum in popular lexicon, it has been the robustness of pioneering research, largely from Japanese scholars, that illuminates empirical links between the PP effects of SY. Tsunetsugu, Park and Miyazaki [13] conducted a novel review representing a didactic integration of various parameters specific to central nervous system (CNS) activity biomarkers; heart-rate variability (HRV), salivary cortisol levels (SCL), immunoglobulin A (IgA) and sense-specific metrics. 

Of the studies included within the PP section, and irrespective of study aims, there was a trend towards small sample sizes, gender and age homogeneity, and skewed ratios of females to males/vice versa, which by either methods of convenience, purpose and/or imparted bias to the research. An overwhelming number of studies included homogenous gender sampling [14,15,16,17,18,19,20,21,22,23,24,25,26,27]. Population demographics specific to gender were unreported in [28,29,30]. Proportionately skewed ratios of male to females and vice versa were identified in these studies [31,32,33,34,35,36]. Studies may have been limited to research participants specific to the student body of the courses and facilities within which the research was designed. The aspects of cultural specificities and sensitivity in research design must be considered when approaching a literature review from a global lens. The methods, tests, and findings of 40 relevant publications expounding upon the PP indices are included in this review. These publications are further assorted into Heart-rate Metrics; Disease States; Autonomic Nervous System Effects; Endocrine Function; and Sense Metrics. The multitudinous reasons for natural environs generating the aforementioned positive qualities have been systematically incorporated into the investigation of the physiological and psychological effects of SY as follows. 

### 3.2. Heart-Rate Metrics

As changes in cardiac function, revealed by cardiac monitoring, are correlational to the physiological effects of stress regardless of environmental setting, it is coherently expected more than one third of the articles reviewed observe not only standard vital signs, including heart rate (HR) and pulse rate (PR), systolic and diastolic blood pressure (SBP/DBP) and ECG interpretation, but also heart rate variability (HRV), as well as left ventricular function and right ventricular function (LVF/RVF). While LVF is attributed to the ability of the left ventricle to perfuse the body, RVF is related to pumping blood to the lungs. HRV is defined as the variation in the time interval between heartbeats and is associated with the activation of the parasympathetic nervous system (PNS) through high frequencies (HF) and the sympathetic nervous system (SNS) through low frequencies (LF).

The trends revealed by SY research over the past 10 years in relation to the cardiovascular and autonomic nervous systems (ANS) appears to have started with basic cardiac monitoring then shifted to the correlation of cardiac monitored data points with the PNS and SNS, and this started the development of a more in-depth study design to research the effects of SY on specific disease states, such as: hypertension (HTN), coronary artery disease (CAD), and chronic obstructive pulmonary disease (COPD). 

According to Kobayashi, Song, Ikei, Song, Kagawa and Miyazaki [18], 625 Japanese males situated in 57 forest-sites and 57 urban-sites across Japan revealed an 80% increase in the parasympathetic indicators of HRV while experiencing the forest setting—physiologically demonstrating forest-viewing was more effective in reducing indicators of stress than in the urban participants. The methods and findings of this large sample study are grounded in some of the earlier pilot studies culled for this review. In Park et al. [37] quantitative study, the sample size was limited to 12 males; however, it was one of the earliest studies to interpret the R-R intervals of the electrocardiogram analyzing pulse rate, in addition to SBP/DBP and LF/HF) components of HRV. This study design [37] has been echoed throughout multiple studies which limit physical activity levels to 20-min for each activity researched in order to control for the cardiovascular effects of physical exertion on each participant [19,22,27,38,39,40,41].

Blood pressure and PR also decreased while in the forest settings compared to the urban settings. In comparison to physical exercise tasks, Lee, Lee, Park and Miyazaki [20] measured HR and BP in relation to synthetic versus organic stimulation. This study revealed both HR and BP decreased in participants after they had completed a garden transplanting task compared with participants’ HR and BP gradual increase throughout a computer performance task. Similar to the findings of Tsunetsugu et al. [40] consisting of 12 males, a study of 17 females over the age of 40 by Ochiai et al. [25] revealed an overall decrease in HR after one day of a “forest-therapy program”. These studies demonstrate cardiovascular benefits for both genders. After controlling for both demographic and socioeconomic factors, Kardan et al. [42] conducted a correlation analyses of data acquired through the Canadian Ontario Health Study. High resolution satellite imagery suggests residents of neighborhoods with a higher density of trees on the streets report less ill cardio-metabolic conditions than do residents of neighborhoods with less trees. Thematically, it is evident the cardiovascular (CV) benefits of SY are apparent regardless of age, gender, socioeconomic background, or previous exposure to a nature setting.

### 3.3. Physiological Disease States

Significant research has revealed the effects of SY and NT on specific physiological disease states, including HTN, CAD, COPD, and Diabetes Mellitus Type II (DMII). In a randomized control trial (RCT) of 24 adults with HTN, Mao et al. [29] found throughout a week-long trip to a nature setting, BP indicators, and CV disease-related pathological factors decreased the activation of the renin-angiotensin system, therefore reducing the workload of the heart and helping to manage the symptoms of HTN. In a RCT of 20 Lithuanian adult patients with CAD, Grazuleviciene et al. [43] found after a week of 30-min sessions in nature, the participants’ cardiac function improved overall. This is a groundbreaking study in there has never been a study prior to their publication that addressed “cardiovascular relaxation and recovery of homeostasis in CAD patients.” Jia et al. [44] found a decrease of perforin and granzyme B expressions accompanied by decreased levels of pro-inflammatory cytokines and stress hormones in 20 patients diagnosed with COPD indicating some of the potential health benefits of SY for individuals living with COPD. Furthermore, in a longitudinal study of 48 adults diagnosed with DMII, Ohtsuka [45] found blood glucose readings declined after multiple SY practice sessions, therefore indicating a significant correlation between SY and the reduction of blood glucose levels. This study is one of the few measuring the effects of SY over time, which indicates further research may be conducted to confirm the long-term effects of SY, not only for its effects on patients with DMII, but for patients with different disease states as well [23,29,43,44,45].

### 3.4. Psychological

**Figure 3:**
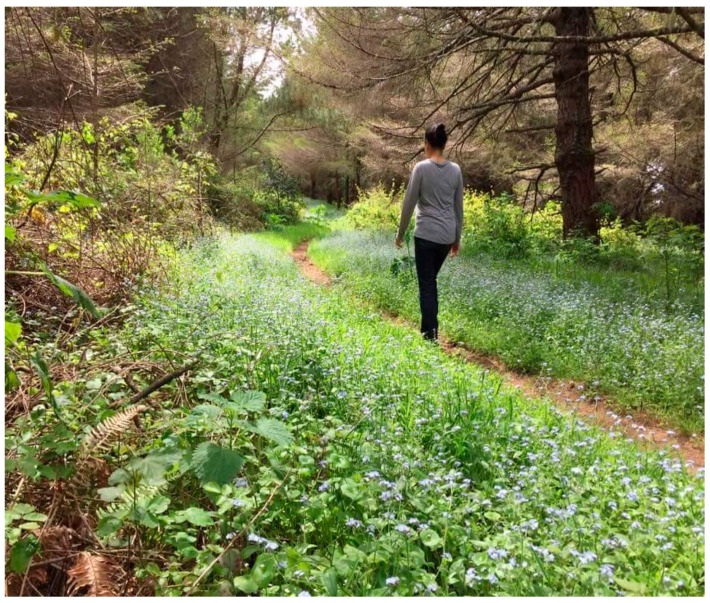
Walking in nature. Permission to publish from CiCi Lee.

Morita et al. [46] noted while SY has been popularized in Japan given the ease of access to forested environments and its’ conscientious governmental recommendations, individuals globally have reduced acute psychological distress from time spent in greenspace (Figure 3).

Furthermore, Morita et al. [46] investigated SY’s effect on 498 Japanese residents suffering from acute and chronic stress. Of these research participants, those suffering from chronic stress states reported the greatest reduction in subjective feelings of hostility, depression and anxiety as a direct result of time spent in the forested environs. Additionally, a RCT reflecting this practice, Sung et al. [47] developed a Cognitive Behavioral Therapy (CBT) based on a FT program, which included educational sessions and guided FB activities for middle-aged men and women diagnosed with Stage I HTN in South Korea. The CBT FT program incorporated elements of meditation and relaxation techniques in chosen forested environments, as well as participant self-reflection and goal setting [47]. The results of which demonstrated a significant decrease in the salivary cortisol (tested as a biomarker of stress), increase in Quality of Life (via the QoL questionnaire) and a decrease in anxiety. However, this study also gleaned a transient effect in the reduction of individual’s manual self-reported BP measures. Self-reported manual BP reports are subject to reliability issues of measurement and bias. Moreover, the lack of additional objective analysis in the short-term forest-environment exposure are identified confounding variables, as well as the fact the participants took prescribed antihypertensive medications throughout the trials in the intervention and control groups respectively [47]. 

A hallmark of SY research has been the investigation of its’ relaxation inducing properties and application for ameliorating psychological distress. Within this review, 12 studies specifically addressed psychological disorders/disease states and relevant comorbid conditions with popular reference to stress and stress related heart disease, emotional distress and chronic depression, alcoholism, sleep disorders, and pain [5,6,26,30,31,32,33,47,48,49,50,51]. Takayama et al. [49] noted the impetus for their SY research stemmed from a growing concern for overworked urban dwellers’ chronic stressors. The results of this comparative study, while limited by sampling bias regarding the subject population consisting entirely of males, indicated a unanimous preference for forest walks versus urban walks based upon data synthesized from participants’ responses to the Profile of Mood States (POMS), Restorative Outcome Scale (ROS) and Subjective Vitality Scale (SVS) pre- and post-intervention. Stress from urban environments caused by surmounting noise and environmental pollution, commuter traffic, financial expenses, increasing tasks, and lack of proximity to FB environs/attributes motivated Park et al. [26] to investigate the connection between psychological distress and greenspace accessibility. This study which included a large, single-sex sample of 168 males between the ages of to 20 to 24 years of age, demonstrated subjects’ preferences for forested environments, specifically in relation to temperature, as demonstrated by participants’ reported lower Predicted Percentage Dissatisfied (PPD) scores related to summer climes within forested environs compared with higher air temperature and heat indexes in urban environments [26]. 

Citing the impact of chronic stress on growing populations with insomnia and poor sleep patterns in Japan, Morita et al. [32] studied forest-walking to induce relaxation and improve general sleep-wake cycles in a population of 71 men and women over the course of three months. Participants reported a statistically significant correlation between increased sleep time (from an average of 365.9 ± 89.4 min to 419.8 ± 128.7 min) post 2-h afternoon forest walks with decreased anxiety. 

McCaffrey, Hansen and McCaffrey [30] investigated garden walking to reduce severity of signs and symptoms of depression in older adults. Participants’ personal stories citing the emotionally healing attributes of the natural surroundings and garden walking paths at the Morikami Japanese Museum and Gardens in Delray Beach Florida, USA inspired the aforementioned researchers. Similarly, Kim, Lim, Chung and Woo [31] investigated the application of a 4-week forest-walking based CBT program for treating clinical depression. Research findings from Kim et al. [31] demonstrated a significant remission rate in the forest walking group at 61% over the traditional psychotherapy hospital-based group at 21%. Kim et al. [31] explicitly cited the work of Australian bush adventure therapy researchers Pryor, Carpenter and Townsend [52] in their pioneering work regarding the connection between time spent in nature and an increase in participants’ health, well-being and emotional confidence. Kim et al. [31] employed a robust research design in so much as researchers incorporated the comparisons of a forest-walking based CBT program (N = 23), a hospital based treatment group (N = 19) and an outpatient control (N = 21). Yet, as with the Pryor et al. [52] research, inherent to the research aim of investigating the previous successes of nature-based therapy [31] is an implicit bias toward the functionality and reliable successes of the research outcomes. 

Given SY practices are relatively innocuous when compared with other more invasive procedures, Chun, Chang and Lee [2] studied FT for patients (N = 59) diagnosed with depression and anxiety (roughly 60–80% of the participants), as well as oxidative stress (roughly 30–50% the participants) that is associated with stroke susceptibility and a positive stroke history. The results of this study, indicated the Beck Depression Inventory (BDI), Hamilton Depression Rating Scale (HAM-D17) and Spielberger State-Trait Anxiety Inventory (STAI) indicate scores were lowered in the post FT intervention group when compared with the control group scores. These results led researchers to recommend FT as a medically viable intervention for the psychological distress associated with chronic illness [2]. Since its’ inception in the 1970s, the STAI has been a hallmark test used to differentiate between participants’ state and trait anxiety, however, its’ brevity and pre-supposed delineation between anxiety-oriented temperaments inspires cause for concern over its’ reliability [53].

Han et al. [3] and Kang et al. [50] focused on chronic widespread pain (CWP) and localized pain in relation to the emotional distresses of coping with the side-effects of intractable pain. In Han et al. [3] psychological indices were measured pre- and post- FT intervention with the BDI and the Visual Analog Scale (VAS) to measure intensity and frequency of CWP. The results revealed statistically significant decreases in pain and associated psychological distress as per the psychometric scales. Whereas, Kang et al. [50] utilized the VAS and the neck disability index (NDI) for chronic neck pain and the McGill pain questionnaire (MPQ) for localized pain, among other measures for physiological indices. Kang et al. [50] measured incidents of painful trigger points in the posterior neck region (TRPs) in the FB with exercise group compared with the FB without exercise, which resulted in reduction by nearly ½ of TRPs in the FB plus exercise group. Widely utilized as a metric for measuring pain, Kang et al. [50] noted the test’s functional role in objectively evaluating participants’ subjective experiences of pain. Researchers noted the popularity of the VAS [51], but further investigation was warranted to determine its’ reliability and validity. The VAS does have a reported test-retest reliability among patients experiencing chronic pain (r = 0.94; *p* < 0.001) in a previous investigation [51], yet, given the subjective nature of pain and uncertain unanimous consensus on pain metrics in international medical literature, this study demonstrated a lack of criterion validity for the VAS.

With regards to human spirituality, Nakau et al. [33] noted in their pilot study involving 22 breast or lung cancer patients, consisting of 4 males (with an average age of 65.3 ± 2.6 years) and 18 females (with an average age of 56.6 ± 11.3 years), that FT can be viewed as an enhancement of spiritual health in cancer patients. Patients in this study [33] were all participating one month or more after undergoing surgery, chemotherapy, or radiation treatment. While patients were not considered to be at risk of life-threatening conditions at the time of study [33], the stress of undergoing treatment for chronic disease was implicated. All patients (N = 22), participated in the integrated FT, horticultural therapy, yoga exercise, meditation and group therapy treatment intervention at the Japan World Exposition (1970) Park in Suita, Osaka prefecture, Japan [33]. The results of this study indicated statistically significant correlations pre- and post-intervention between green environments and individuals’ experiences of self-realization, increased emotional health and integrative well-being, as measured by the Japanese version of the Functional Assessment of Chronic Illness Therapy-Spiritual Well-Being Scale (FACIT-Sp), QoL questionnaire, Cancer Fatigue Scale, POMS and STAI, in addition to physiologic measures of NK activity [33]. 

### 3.5. Autonomic Nervous System Effects

Research on the cardiovascular effects of SY have precipitated a trend to discover how FB affects the ANS. At the level of the central nervous system (CNS) alone, marked changes in cerebral activity have been identified. Joung et al. [28] designed novel research to investigate identified specific anatomical cortices within the brain that vary in stimulation to both forest and urban areas. Activity in the prefrontal cortices of the forest-area group participants were significantly lower than that of the city-area group participants in the “after walking” through their randomly assigned locations. This decrease in activity suggests a strong correlation between nature settings and ANS activity [28]. Research has shown peak HF levels can be seen within 5–7 min of each nature experience, which demonstrates not only will SY benefit the health of the ANS, but that positive HF components of HRV are evident within minutes of forest immersion [9]. Regarding other physiologic indicators of stress, Mao et al. [24] conducted a quantitative RCT that demonstrated after short periods of time in nature, measures of malondialdehyde (MDA) concentrations, cytokine production, serum cortisol, testosterone, and lymphocytes decreased. Universal findings revealed LF components were significantly lower in the forest areas than in the city areas, while HF components of HRV tended to be higher in the forest than in the city and therefore these research findings are important for further physiological research and the effects of SY [17,18,20,23,24,25,27,52].

### 3.6. Endocrine Function

This section, a further investigation into the physiological and psychological effects of SY or NT via the physiological metrics relevant to endocrine included 11 publications. These resources specifically measured the effects of SY or NT on specific physiological and psychological indicators of stress via measures of salivary cortisol (sCort), and/or emotional indicators of health and well-being [1,17,24,25,26,27,34,36,40,47,48,49,52,53]. Kobayashi and Miyazaki [53] studied baseline cortisol measures in 267 healthy male students from The University of Chiba, Japan, with the aim to compare measures in future SY studies. In Largo-Wright et al. [48] researchers deduced a correlation between increased contact with nature and decreased stress levels and generalized health complaints in office workers at a Southeastern university in the U.S. via the Nature Contact Questionnaire (NCQ), The Perceived Stress Questionnaire (PSQ), and a health behavior assessment derived from contact with the outdoors, over other types of contact with nature, such as indoor plants [48]. Additionally, practices of SY have demonstrated statistical significance in lowering blood-glucose. In a 2012 longitudinal trial, researchers from Hokkaido University, Japan, demonstrated forest-walking reduced blood glucose levels in 48 Type 2 diabetic patients [34]. A total of 48 participants, 16 males and 32 females, with a mean age of 66.8 years and diagnosed with DMII, walked for distances of 3 to 6 km nine times per week over a period of 6 years. There was no statistically significant difference between the subjects’ glucose levels, or HbA1c levels between the shorter and longer walks. However, averages of both groups’ blood glucose levels pre and post-forest walks declined by 79 ± 10 mg/dL and 76 ± 7 mg/dL respectively [34]. 

In the pilot study with a cross-over experimental design, investigators [52] addressed the impact of participants (N = 15) exposure to four urban and natural environments on physiological and psychological stress matrices. The study [52] utilized sCort and salivary amylase (sAA) as metrics, which the authors note via the work of Engert et al. [54] have been significantly reliable physiological biomarkers for stress. Additionally, Beil and Hanes [52] obtained data from questionnaires measuring participants’ experience of stress via the Perceived Stress Scale (PSS), Perceived Restorativeness Scale (PRS), Subjective Stress Scale (SSS) and susceptibility of affective connections to natural environments via the Environmental Identity Scale (EID), pre- and post-intervention, which included a 20-min exposure to 4 different environments ranging from the “mostly built” to the “mostly natural.” The participants’ sAA and sCort levels respectively peaked after exposure to the urban environments, while levels were within normal range post exposure to the natural environments, which also correlated with participants’ subjective impressions of stress [52]. The EID, while relatively novel, was noted to have been previously tested for its’ effectiveness in ascertaining subjects’ general health and welfare status in response to the environment [52]. 

### 3.7. Sensory Metrics (SM)

Fisher [55] illuminated an emerging interest in FT practices for psychological and physiological healing. By interviewing a proponent of FT, Fisher described the growing trend of individuals restoring native tree habitats with the dual purpose of environmental stewardship and psychological welfare of the persons involved in the process; paralleling the sentiments of many SY researchers [9] and supporting the Biophilia hypothesis [8]. Furthermore, Stigsdotter [36] conducted a case-study that followed up on survey driven data collected from a 2005 Danish Health Interview of 10,125 adult males and females and, results revealed significant positive correlations between access to green-space within 1 km, self-perceptions of stress and general health and well-being. 

A subset of publications explicitly focused on sensorial stimuli, as a function of the effects of SY, which included time spent in forested outdoor environments, interactions with elemental aspects of natural environments and laboratory settings. Tsunetsugu et al. [13] synthesized evidence from physiological and psychological indices into subcategories, which exemplified the biomechanics of SY impact on the five senses. Eight key publications specific to SM were incorporated within this review, including: the metrics of olfaction [14,16], tactile stimulation [19], and visual stimulation, or neurological response [11,15,28,56,57].

Research included within the SM theme invariably measured nervous system activity and emotional response of participants in relation to experiencing authentic aspects of forested environments by comparative means. Igarashi et al. [56] studied participants’ HRV, as an indicator of PNS activity in 48 high-school students viewing real vs. artificial pansies, the results of which illustrated a stronger correlation between relaxation with the real pansies versus the silk flowers. This was represented by a significant decrease in the ratio of LF/HF HRV, and subjective analysis of students’ self-perception of relaxation indicating preference towards real flowers. Furthermore, Igarashi et al. [11] analyzed right and left prefrontal cortical activity in terms of cerebral blood-flow, and hemoglobin concentration changes via Near Infrared Time Resolved Spectroscopy (NIRS), which was measured in units of micro-meters (µM), according to the wavelengths observed. This was performed before and after participants were subjected to four visual conditions (real dracaena plants, images of dracaena plants, cardboard boxes and images of cardboard boxes) for timed intervals of 0–3 min each. Notably, µM concentrations were higher in participants viewing the actual dracaena plant stimulus for 3 min versus the pictorial sample for the same amount of time in right and left prefrontal cortical areas respectively [11]. Joung et al. [28] also utilized NIRS to determine µM levels pre-posttest upon participants viewing actual forested versus urban areas. Counter to the aforementioned Igarashi et al. [11] study, participants demonstrated increased subjective measurements of relaxation correlated with decreased µM concentrations and prefrontal cortical activity, and increased feelings of calmness from the forested site vs. urban site [28]. This may indicate not only are aspects of the natural environment optically stimulating as illustrated by Igarashi et al. [11] but they may also require less executive functioning as demonstrated by Joung et al. [28]. Mutual feelings of “calmness” were derived from each study. 

Igarashi et al. [15] looked at comparisons between participants viewing images of kiwifruit orchards and specific urban areas in Japan. The POMS and HRV were used to evaluate participants’ responses, which demonstrated moderate increases in PNS activity and feelings of “relaxation and calmness” when viewing the orchard versus an urban setting. Subjective measures of stress reduction have been consistently apparent in the studies focused on visual stimulation regardless of physiological indices. In Tsutsumi et al. [57], researchers aimed to investigate and compare participants’ relaxation states before and after viewing visual footage of forested landscapes, and comparatively, seascapes. Measures of HRV, results from POMS questionnaires, and Bispectral Index System analysis (measured brain activity via electrode placement) allowed researchers to determine participants’ sleep-wake states while comparing brain wave activity. Findings indicated significant decreases in HR, greater relaxation in post-intervention analysis in both groups, with the forest-viewing group demonstrating the greatest difference in relaxation-states across all measures [57]. These studies give statistically significant credence to the notion visual stimulation by aspects of forested environments reduces stress and increases a general sense of well-being in various study populations. Furthermore, these studies serve as templates to be integrated into therapeutic practices as suggested by Tsutsumi, et al. [57]. 

Koga and Iwasaki [19] investigated the potential for foliage-based tactile stimuli to induce relaxing effects that have been demonstrated via SY based field experiments. These researchers [19] utilized NIRS to detect cerebral blood-flow and the Semantic Differential (SD) model to determine emotional responses in participants’ experiences with touching leaves versus other non-natural substances, such as plates of metal and fabrics. As noted by Park et al. [58], laboratory-based research on the physiological and psychological effects of SY has been paramount. Furthermore, the Koga and Iwasaki [19] study revealed statistically significant correlations between touching natural substances, such as tree-bark, and incidents of decreased blood pressure. Moreover, these findings are associated with an increase in participants’ subjective feelings of calmness. However, despite the clarity in exposition, researchers didn’t identify the reliability and validity of the SD method utilized in the study. As with several SY studies, specific to psychological indices, self-reported measures in the form of questionnaires, such as the SD method, provided a bulk of the data. Therefore, leaving a question about the consistency, validity and reliability of the reported psychological outcomes. 

Previous research regarding the effects of SY explored elements of olfaction as a sense metric relevant to the biological effects of experiencing forested environments. Tsunetsugu et al. [13] noted in their review, phytoncides, or volatile organic chemical compounds released from plants and trees have previously been associated with the effects of SY. Furthermore, Li et al. [63] described how the scent derived from phytoncides of 13 different tree species (phytoncides are unique to each specie and serve as a critical communication pathway between trees classified under the same genus) increased human Natural Killer Cell activity and decreased adrenaline in the FT intervention group of the study comparing urban and forested environments on human immune and stress function. Ikei et al. [16] studied the impact of α-pinene, a phytoncide from Japanese cedar wood (which is notably ubiquitous in forested areas of Japan) on ANS function via HRV indexes and the SD method. The results of which indicated an increase in PNS activity and a decrease in heart rate [16]. 

Jo et al. [14] focused research efforts on “floral scent”, specifically Japanese plum blossoms, utilizing NIRS, HRV, POMS, and the SD as the physiological and psychological indices. Researchers created a unique apparatus for metering the floral and control scents, which involved a polypropylene pressurized bag with a constant flow of controlled air to be inhaled by participants. This novel approach controlled for many potential confounding factors given the ubiquity of various scents in laboratory settings. Utilizing multichannel NIRS enabled researchers to investigate the effects of olfactory stimulation on 47 localized neurological regions corresponding to areas noted for emotional, judgement, motor control, memory, somatosensory, cognitive, visual, auditory, and speech functions [14]. The literature expounding upon sense-metrics pertaining to the effects of SY illuminates a thorough pursuit of specificity and ingenuity.

Following a systematic review of 25 articles from databases including, but not limited to PubMed, EMBASE, CINAHL, and PsychINFO, Bowler [1] found each study suggested natural environments may have direct and positive impacts on humans’ overall well-being. Bowler recognizes it is difficult to truly separate the raw effects of experiencing nature from confounding factors, such as physical activity performed, previous exposure to nature, as well as an optimal time frame for these effects. However, Bowler and every author mentioned throughout this literature review stress the aim to encourage research on the health benefits associated with the practice of SY. While research in primarily Japan and China has shown a positive impact of SY on both the physiological and psychological structures throughout the human body, it also calls for Western cultures to incorporate elements of the SY practice, so as to demonstrate compatible results world-wide for both patients and their healthcare providers [1].

### 3.8. Conceptual Frameworks

#### 3.8.1. Nature Therapy

During the review of the literature, existing conceptual frameworks emerged that may be applied to SY practice and NT research. The first conceptual framework (CF) is Thomson’s “vis medicatrix naturae,” otherwise known as, “the innate ability of the body to heal itself” as presented in Logan and Selhub’s review of the effects nature has on the human brain [59]. Thomson posits the healing power associated with nature is directly connected with an individual’s intentional contact with “animate and inanimate” aspects of the outdoors, such as touching the bark of a tree. While recognizing today’s citizens’ increased use of technology, exposure to air pollution and the associated increased stress responses, Logan and Selhub [60] present questions based on Thomson’s framework. For example, “What might be an appropriate “dose” (duration and frequency) of nature contact to reduce stress?” “Are certain types of activities (e.g., gardening, walking in forest settings, contemplating in an urban park) more effective than others?” [60] Future directions for research, global urban planning and architecture, and policy making may be based on Thomson’s framework of “vis medicatrix naturae.” Furthermore, the research conducted by Selhub and Logan directly points to the health benefits associated with natural environments and may be parallel to the practice of SY and NT. 

#### 3.8.2. Psychological Underpinnings

The second CF is by Berger [61]. Berger presents a novel, autonomous and independent NT framework that serves as a model to support art and drama therapy. Within this theoretical and applied framework, which is considered “integrative” because it takes place in nature and serves as a part of the human healing process is the self- “reflexive” process that includes personal experiences. The NT model and theory are supported by past evidence derived from Gestalt psychology and the narrative research approach mixed with traditional “ritual” foundations. This novel theory attempts to put a spotlight on modern individuals’ detachment from nature, absence from community engagement and spirituality through a psycho-eco-social lens. Berger purports individual’s personal estrangement from nature, lack of involvement in community affairs and spirituality as being main factors influencing the modern-day spread of loneliness, depression, anxiety, low self-esteem and detachment. SY practice and NT research may be grounded in Berger’s CF that recognizes the healing natural forces, resilience and recovery associated with nature. 

The third CF is from a Threshold Concept and Transformational Learning perspective [62]. The practice of SY assisted by trained nature and forest therapy guides leads individuals into a “liminal” space. In this “liminal” space, also known as an “in-between” human state or “suspended state of partial knowing,” the healing properties associated with SY are purportedly activated [62]. During the “liminal” phase, a person integrates, discards and experiences an “ontological shift” and then experiences “transformation” and a “changed discourse,” known as a “post-liminal phase.” The individual may experience a “pre-liminal” space in nature and may vacillate between old and emergent thoughts that may be disruptive. However, once in the “liminal” psychological space, the individual experiences a sense of calm and mastery. The immersion into nature may lead to a transformative way of knowing and understanding the self. These noteworthy concepts may serve as foundations for future research studies.

### 3.9. Limitations

Limitations of this review include the biases among the authors of the studies and articles culled, as well as the conceivable restrictions of SY as an evidence based practice within the traditional principles of Western medicine. While search criteria for the articles remained consistent across all database searches, publication bias must be acknowledged as most of the studies reviewed demonstrate a positive correlation among SY practice and NT with favorable physiological and psychological outcomes. In addition, original study sample sizes were often limited to less than 20 participants, with the inclusion criteria of primarily of healthy, young, male university students, making results difficult to generalize across entire populations. Other limitations within the studies include their inability to distinguish physical and psychological effects purely based on the participants’ surroundings versus the participants’ level of activity while present in either an urban or nature setting. Most studies offer little distinction among senses used, and which, if any, have a greater influence on positive or negative outcomes. While the current research has continued to trend toward the benefits of SY and NT on specific disease states and diagnoses, it has primarily focused on the short-term effects of the practice of SY and NT with little research to indicate the longevity of its benefits. This concept of permanence relates not only to the amount of time spent in a nature setting for short-term optimal results within a study timeframe, but the participants’ previous relationship with nature throughout their lifetime, and how a priming bias may influence the amplitude and frequency of corporeal effects. SY as a therapeutic practice to be exemplified by healthcare providers and recommended to their patients includes the limitations of theoretically defining SY for clinical use, the social and economic determinants of health which limit access to natural environments, and the correlation between the ever changing diversity of nature itself and the unpredictable physiological and psychological responses it may induce within the human body as noted by the Biophilia Hypothesis [8]. 

## 4. Discussion

### 4.1. Overview of Health Benefits of SY and NT

In general, from a physiological perspective, significant empirical research findings point to a reduction in human heart rate and blood pressure and an increase in relaxation for participants exposed to natural GS [13,40]. Even research involving the use of nature videos of the forest or the ocean have the same physiological effects [60]. From a qualitative and psychological perspective, Danish participants reported a sense of safety, calm and overall general wellbeing following exposure or engagement with nature [63]. South Korean participants with a known alcohol addiction and high pre-test scores of depression benefited more from the Forest Therapy Camp than participants with lower pre-test scores of depression and alcohol abuse [35]. Differences in culture, gender, education, marital or economic status were not associated confounding factors in many of the empirical studies. Overall, our review of the literature, as illustrated in Table 1, points to positive health benefits associated with SY and NT while confounding factors were clearly identified by the researchers. 

### 4.2. Implications for Future Research

The aims of this state-of-the-art review are to showcase and elucidate the existing research on the effects of the practice of SY and NT on human physiological and psychological systems, existing frameworks and sense metrics in order to assist researchers in conducting future empirical research to substantiate, gain a better insight and enrich the scholarly literature. Longitudinal research is recommended to investigate the powers of nature as a healer and preventative element in today’s modern/technological driven world over a long period of time. Proposed thoughtful and well-designed research would include both quantitative and qualitative approaches to identify specific SY and NT factors that influence disease and health promotion in Western Cultures. Furthermore, a systematic review of the current literature would honor the scholarly work completed to date and provide a higher level of evidence for the practitioner considering SY and NT as EBP interventions. 

### 4.3. Implications for Future Healthcare Practice

This in-depth review illustrates, honors and supports the increased awareness of the positive health-related effects (e.g., stress reduction and increased holistic well-being) associated with humans spending time in nature, viewing nature scenes via video, being exposed to foliage and flowers indoors and the development of urban green spaces in large metropolitan areas worldwide. Not only valid and reliable psychometrics have been implemented, but valid and reliable physiological measurements have been used to show significant and potentially healing and health promoting effects. Furthermore, physiological and psychological research, based on sound NT frameworks and hypotheses is needed in the areas of healthcare professional/student stress-reduction and life balance [64,65]. 

Healthcare professionals and educators may turn to the SY and NT literature for simple, affordable and enjoyable complementary interventions to reduce stress, anxiety, and depression symptoms and enhance joy, relaxation, overall sense of well-being and balance in life. The founder and faculty member of the Association of Nature and Forest Therapy Guides and Programs, Amos Clifford, states the organization’s mission is to integrate FT into healthcare systems [62]. Moreover, the profession of nursing and medicine has moved toward an integrative approach to healthcare. The third integrative nursing principle: “Nature has healing and restorative properties that contribute to health and well-being” supports the health benefits associated with the practice of SY and NT and serves as a part of the integrated healthcare model [66]. Furthermore, SY as a healing and restorative modality may support the clinician’s and patient’s whole-being while promoting a sense of peace, dignity and comfort. These ideas are supported by Watson’s Carative Processes [67], specifically *Process Eight*: *Creating healing environment at all levels, whereby wholeness, beauty, comfort, dignity, and peace are potentiated.*

## 5. Conclusions

Advancements in complementary and alternative medicine (CAM) are indicative of a time in history when researchers and practitioners are willing to look at how humans evolved over the past 6- to 7-million years. When one ponders humans existing less than 0.01% of the species’ history in modern surroundings and the other 99.99% of the time living in nature, it is no wonder some humans yearn and are drawn back to where human physiological/psychological functions began and were naturally supported. The Biophilia Hypothesis [8] supports SY and NT because it is steeped in the idea humans have an inner biological attraction to nature and its importance in our human development. Moreover, psychologically and spiritually speaking, humans intuitively know the relaxing, soothing and “awe” effects of being in or viewing forests, plants, flowers, urban green spaces, parks and natural wooden materials [68,69]. The mind-body-spirit experience associated with SY is for all humans and may be accomplished in various documented ways as illustrated in the novel review. These methods are supported by current scientific data, history and personal experiences reported over time. The practice of SY and NT are ontological realism and offer humans an authentic way of healing and health prevention for the mind, body and spirit [70,71,72]. How might we continue to encourage this health-promotion method and demonstrate scientifically and intuitively in order to add to EBP and global healthcare systems? 

## Figures and Tables

**Table 1:** Characteristics of selected studies and supporting evidence.

Study	Country	Population	Sample	Setting	Aim & Design	Findings
Bowler [1]	UK	Articles were culled from PubMed, EMBASE, CINAHL, PsycINFO, Web of Science, SPORTDiscus, ASSIA, HMIC Data, LILACS, UK Natl. Research Register archives, TRIP database, UK Natl. Lib. for Health, Index to Theses Online, Directory of Open Access Journals, Economic and Social Data Service, Database of Promoting Health Effectiveness Reviews, Trials Register of Promoting Health Interventions, Cochrane Collab., Campbell Collab.	Article total = 25. Studies that met the review inclusion criteria included crossover or controlled trials, which investigated the effects of short-term exposure to each environment during a walk or run. Including ‘natural’ environments, such as public parks and green university campuses, and synthetic environments, such as indoor and outdoor built environments.	Centre for Evidence-Based Conservation at the School of the Environment and Natural Resources, Bangor University, Bangor, Gwynedd, United Kingdom.	Systematic review to collate and synthesize the findings of studies that compare measurements of health or well-being in natural and synthetic environments. Effect sizes of the differences between environments were calculated and meta-analysis used to synthesize data from studies measuring similar outcomes. PP	The studies suggested that natural environments may have direct and positive impacts on several aspects of health and well-being.
Chun [2]	Korea	Chronic stroke patients recruited from a stroke welfare center in the Republic of Korea. Of those included: 31 patients had a history of cerebral infarcts, and 28 with a history of intracerebral hemorrhage.	N = 59; 40 men, 19 women; 60.8 ± 9.1 years of age with an age range of 36–79 years.	Settings included a recreational forest area in Gyenggi-do, Republic of Korea. The urban group stayed in a hotel Gyenggi-do in the Republic of Korea.	Assessment of forest therapy effectiveness for treating depression and anxiety in patients with chronic stroke by using psychological tests. This study measured reactive oxygen metabolite (d-ROM) levels and biological antioxidant (BAPs) potentials associated with psychological stress. Patients were randomly assigned to a forest group (recreational forest site) or urban group (staying in an urban hotel). Scores on Beck’s Depression Inventory, Hamilton Depression Scale, and the Spielberger State Trait Anxiety Inventory were analyzed. PP	Forest groups had BDI, HAm-D17 and STAI scores were significantly lower following treatment. BAPs were significantly higher than baseline. Urban group STI scores were significantly higher following treatment. Forest therapy is beneficial for treating depression and anxiety symptoms in patients with chronic stroke and may be useful in patients who can’t be treated by standard pharmacological or electroconvulsive therapies.
Han [3]	Korea	Employees of a public organization providing building and facilities management services in Seoul Metro area, all of whom were diagnosed with Chronic Widespread Pain (CWP).	N = 61; 35 females and 26 males; randomly assigned to the either the experimental forest therapy group (n = 33), or the control group (n = 28).	Forest therapy intervention took place at a campsite at the Saneum Natural Recreation Forest in Yangpyeong county of Gyeonggi Province. Additional assessments were taken at the Inje University Seoul Paik Hospital in the urban environment.	To explore the effects of a 2-day forest therapy program on those with chronic widespread pain. Measures assessed included the following: pre- post heart rate variability, natural killer cell, self-reported pain, depression level and health related quality of life. PP	Forest therapy participants reported significant decreases in pain, depression and increased QOL. Forest therapy is an effective intervention to relieve psychological and physiological pain.
Anonymous [4]		Supporting material	NA	NA	Shinrin Yoku. A website describing the practice of SY and programs offered for forest guide training.	NA
Williams [5]		Supporting material	NA	NA	An article presented in the National Geographic magazine about the effects of NT.	NA
Kaplan [6]		Supporting material	NA	NA	A book about Kaplan’s Attention Restorative Hypothesis.	NA
Ulrich [7]		Supporting material	NA	NA	An article about Ulrich’s Stress Reduction Hypothesis.	NA
Kellert [8]		Supporting material	NA	NA	A book explaining the Biophilia Hypothesis.	NA
Song [9]	Japan	Researchers culled articles from the Pubmed database using various keywords.	Article total: 52	NA	Literature review aimed to objectively demonstrate the physiological effects of NT. Reviewed research findings in Japan related to the green space, plants and wooden material and the analysis of differences that arise therein. PP	Researchers elucidated various scientific data, which assessed physiological indicators, such as brain activity, autonomic nervous activity, endocrine activity, immune activity are accumulating from the field and lab experiments. NT will play a significant role in preventative medicine in the future.
Sifferlin [10]		Supporting material	NA	NA	A Time magazine article about the effects of living and interacting in green spaces. Results indicate people are more energetic, in good overall health and have more of a sense of meaningful purpose in life.	NA
Igarashi [11]	Japan	Female students from the University of Chiba, Japan, deemed healthy at the time of the study.	N = 18; adult female university students with a mean age of 21.6 ± 1.5 years.	Artificial climate chamber in a laboratory of the Center for Environment, Health, and Field Sciences, Chiba University, Japan.	Quantitative study was to determine if images of natural objects elicited similar neural responses (activation of the prefrontal cortex) as those brought about with the interaction of real objects. Physiological measurements were performed in an artificial climate chamber maintained at 25 °C with 50% relative humidity and 300 lux illumination. For foliage plants three dracaena plants (*Dracaena deremensis*) were used. Oxy-hemoglobin concentrations in the prefrontal cortex were assessed with time-resolved near-infrared spectroscopy. SM	Subjects viewing actual live plants had significantly increased oxy-hemoglobin concentrations in the prefrontal cortex. Subjective ratings of “comfortable vs. uncomfortable” and “relaxed vs. awakening” were similar for both live and artificial plants. Results were significant for the benefits for urban, domestic and workplace foliage.
Livni [12]		Supporting material	NA	NA	The Japanese practice of ‘Forest Bathing’ as scientifically proven to improve your health.	NA
Tsunetsugu [13]	Japan	N/A	Author-researchers culled articles for this literature review from a studies rooted in physiological data, data collected from field experiments in forest settings, laboratory settings, and studies categorized into sub-themes specific to the five-senses. Exact literature search methodology and total number of articles culled unknown.	N/A	To investigate the physiological effects of Shinrin-yoku according to specific themes centered on the health applications of FB. The authors reviewed previous physiological experiments with trials in forests and laboratory settings, to determine the physiological effects on individuals from exposure to forests and elements of forest settings. Metrics investigated included: physiological measurements of central nervous activity, autonomic nervous activity, and biomarkers reflecting stress response. PP	Author-researchers summarized the separate elements of forests in terms of the five senses, and provide contribution to effects of Shinrin-yoku within the framework of the “Therapeutic Effects of Forests” project.
Jo [14]	Japan	Participants comprised Japanese male graduate and undergraduate students at Chiba University, recruited from landscape and horticulture programs.	N = 26; males aged early to mid- twenties mean age 24 ± 1.8 years.	Chiba University in a screened room, such that the participants were blinded to the observers.	Quantitative. Controlled trial without randomization. The aim of this study was to elucidate how floral fragrance could impact human health; specifically, the psycho-physiological responses to the floral scent of the Japanese plum blossom. Changes in cerebral activity were measured by multichannel near-infrared spectroscopy. Pulse rate, heart-rate variability and arterial blood pressure were taken. The brief-form Japanese version Profile of Mood States questionnaire (POMS) tested for psychological stress. SM	Sensory stimuli from plants may reduce stress and provide a general sense of wellbeing among this population. Hypothesis was supported by the data.
Igarashi [15]	Japan	Seventeen Japanese adult females were recruited from a population living within the urban suburbs of Kashiwa in the Chiba Prefecture of Japan. All were deemed healthy prior to the experiment.	N = 17; Adult Japanese females with a mean age of 46.1 ± 8.2 years.	Kiwifruit orchard adjacent to the Center for Environment, Health and Field Sciences, Chiba University, Japan.	Physiological and psychological relaxation effects of viewing kiwifruit orchard landscapes in summertime in Japan were investigated. Quantitative, randomized controlled trial wherein subjects viewed a kiwifruit orchard landscape or a building site (control) for 10 min. Intervals. HRV and HR were measured continuously. Modified semantic differential method and short-form Profile of Mood States (POMS) were determined. SM	Significant increase in PNS activity and marginally significant decrease in HR and an increase in comfortable, relaxed and natural feelings and a significant improvement in mood states.
Ikei [16]	Japan	Female students from the University of Chiba, Japan, deemed healthy at the time of the study.	N = 13; adult female university students with a mean age of 21.5 ± 1.0 years.	Center for Environment, Health and Field Sciences, Chiba University, Japan.	Quantitative controlled trial without randomization was to determine the effects of olfactory-stimulation of the alpha-pinene (a volatile compound in Japanese cedar wood) on autonomic and parasympathetic nervous system activity. Measures were taken at 30 s before and 90 s during/after smell admin. HRV and HR were measured. HR SM	Olfactory stimulation by a-pinene significantly increased the High Frequency measure of HRV, which is associated with parasympathetic nervous activity, and decreased HR overall—these are signs of increased physiologic relaxation.
Kobayashi [17]	Japan	The study consisted of 456 Japanese male students at the University of Chiba, Japan, deemed healthy at the time of the study.	N = 456; Males aged 20 to 29 years old (mean, 21.9 ± 1.6 years).	Experimental design and procedures took place at the Forestry and Forest Products Research Institute and Center for Environment, Health and Field Sciences, Chiba University, Japan.	To deduce and present the “normative values,” or “reference” range of heart rate variability (measured in 417 young male students), and salivary alpha-amylase in 430 “healthy” male students from Chiba University with an emphasis on the distribution and reproducibility of the values. Measures within this quantitative study included: short-term HRV; beat to beat HR recorded at 2 min intervals with portable/wearable HR monitor Salivary alpha-amylase measurements taken before breakfast (6:30 to 7:30 a.m.) after subjects sat “resting” 1 min. PP	Results suggested a relatively small correlation between HRV and salivary alpha-amylase. This study is mostly indicative of intra-individual variability in measures. Provides example of metrics we can use in our study as well as “normative” values.
Kobayashi [18]	Japan	The population consisted of in 267 male students from The University of Chiba, Japan, deemed healthy at the time of the study.	N = 267: Males with a mean age of 21.7 ± 1.5 years.	Chiba University’s research labs and additional laboratory studies performed at the laboratories of SRL Inc. in Tokyo, Japan.	Quantitative study aimed to specify the normal salivary cortisol levels, and reference ranges in subjects at University of Chiba, as a relatively innocuous biomarker for stress levels during the mornings on two consecutive days, which were analyzed by radioimmunoassay. Quantitative. Saliva collected before breakfast, appx. 20–40 min after awakening (6:30–7:30 a.m.) and again before participants brushed teeth. Each subject rested for 1 min in a sitting position before saliva collection. Measures were repeated the following day. PP	Consistency and reliability (“distribution characteristics”) of salivary cortisol measures were reported to be steadier in the morning samples ~30–45 min after waking.
Koga [19]	Japan	Japanese male students from The University of Chiba were recruited for this study.	N = 14; Males with an age range of 21–27 years.	Laboratory rooms at The University of Chiba, Japan.	Quasi-experimental study was to gauge the feeling elicited by (with eyes closed), touching four different “tactile” samples: a plate of aluminum, a piece of velveteen, leaf of natural Epipremnum aureum, and an artificial resin-made leaf, for about ~120 s. Measures included pre and posttest psychological and physiological indices, Cerebral Blood Flow (hemodynamics) measured via near infrared spectroscopy (NIRS; NIRO-300; Hamamatsu Photonics, JAPAN); measured pre and post stimulus. Psychological data were acquired using a semantic differential questionnaire. SM	Participants successfully reported feeling a measurable sense of “calm” when touching natural plant material, as opposed to the other materials
Lee [20]	Japan	Male students from the University of Chiba, Japan.	N = 24; Japanese males with a mean age of 24.9 ± 2.1.	Settings included laboratory rooms at The University of Chiba, Japan.	To examine the psychological and physiological benefits of interaction with indoor plants vs. computer tasks. Researchers implemented a quantitative crossover experimental design. Participants were randomly distributed into 2 groups (n = 12 plants; n = 12 computer task). PP	Feelings during the transplanting task were different from that during the computer task. Feeling more comfortable, soothed, and natural after the transplanting task Sympathetic activity increased over time during the computer task but decreased at the end of the transplanting task. Diastolic BP lower after transplanting task.
Lee [21]	Japan	Twelve young Japanese male adults were recruited from local universities. At the recruitment stage, those who had past or current mental disorders, and those with cardiovascular or allergic diseases were screened. Those who were habituated to smoking or drinking were excluded. The adults who participated in the study had a mean age of 21.2 years [standard deviation (SD) 0.9].	N = 12; Japanese males with a mean age of 21.	Field experiments were performed at two different sites (forest and urban) in Hokkaido Prefecture, Japan. The forest site was characterized by broad-leaved deciduous trees and was located in Tsurui Village. The urban site was a typical commercial area situated in the town of Kushiro.	To provide scientific evidence supporting the efficacy of FB as a natural therapy by investigating its physiological benefits using biological indicators in outdoor settings. 3 days 2-night study. Physiological responses as well as self-reported psychological responses to forest and urban environmental stimuli were measured in real settings. PP	Results of each indicator were compared against each environmental stimulus. HF power analysis, which reflects the activity of the parasympathetic nervous system, significantly higher values were obtained for forest stimuli than urban stimuli. Additionally, LF/HF ratio values of HRV, which mediate the activity of the sympathetic nervous system, were significantly lower in the forest than at the urban site.
Lee [22]	Japan	Male students ages 21–22 at Chiba University in Japan were recruited to participate in the walking programs. All were deemed healthy at the outset of the trials.	N = Japanese males with a mean age of 21.1 ± 1.2 years.	Field experiments were performed at four different sites in Japan including: Yoshino Town in Nara Prefecture, Akiota Town in Hiroshima Prefecture, Kamiichi Town in Toyama Prefecture, and Oita City in Oita Prefecture. Data analysis performed at The University of Chiba, Japan.	Quasi-experimental study aimed to compare the effects of a forest walking therapy program with an Urban walking program over 2 consecutive days to determine the cardiovascular relaxation indices. Walks included 12–15 min of self-paced walking in forest (4 sites selected throughout Japan) and urban (the control) environments. HRV measured with a portable ECG w/in 1 min intervals. 4 psychological questionnaires delivered: semantic differential (SD) techniques. Japanese version of the Profile of Mood States (POMS). Anxiety levels studies with Spielberger State-Trait Anxiety Inventory (STAI). PP	Cardiovascular relaxation was noted with the forest walking therapy program, but not as with the urban control -specifically the differences in HRV and BP within the two exposures. Psychological tests are concurrent with these findings.
Li [23]	Japan	Male adult subjects were selected from four large companies in Tokyo, Japan. Subjects having infectious disease, utilizing immunosuppressive drugs and/or other relevant medications were ruled out. Subjects were deemed healthy at the time of the trial.	N = 12; Males aged 35–56 years, with a mean age of 45.1 ± 6.7.	Various forested and urban locations across Japan. Specifically, the FB groups experienced three different forest areas in Agematsu town in Nagano prefecture of northwest Japan. Whereas the city group experienced Nagoya city located in Aichi prefecture in the center of Japan.	To study NK activity in both forest and urban environments. Twelve healthy male subjects, age 35–56 years, experienced a three-day/two-night trip to forest fields and to a city, in which activity levels during both trips were matched. On day 1, subjects walked for two hours in the afternoon in a forest field; and on day 2, they walked for two hours in the morning and afternoon, respectively, in two different forest fields; and on day 3, the subjects finished the trip and returned to Tokyo after drawing blood samples and completing the questionnaire. Blood and urine were sampled on the second and third days during the trips, and on days 7 and 30 after the trip, and NK activity, numbers of NK and T cells, and granulysin, perforin, and granzymes A/B expressing lymphocytes in the blood samples, and the concentration of adrenaline in urine were measured. Similar measurements were made before the trips on a normal working day as the control. PP	Phytoncide concentrations in forest and city air were measured. The FB trip significantly increased NK activity and the numbers of NK, perforin, granulysin, and granzyme AlB-expressing cells and significantly decreased the concentration of adrenaline in urine. The increased NK activity lasted for more than 7 days after the trip. In contrast, a city tourist visit did not increase NK activity, numbers of NK cells, nor the expression of selected intracellular anti-cancer proteins, and did not decrease the concentration of adrenaline in urine.
Mao [24]	China	Male University students deemed healthy at the time of the trials without documented history of physiological or psychiatric disease and/or disorder.	N = 20; Male age 20.79 ± 0.54 years.	Locations included the Wuchao Mountain Forest in Hangzhou, Zhejiang, China and the urban, downtown district of Hangzhou, China.	Quantitative randomized controlled trial was to measure the effects of forest-bathing for short periods of time on overall human health using a variety of metrics. To investigate potentially positive effects of FB on the subject’s health from the standpoint of pathophysiological metrics. Subjects were randomly divided into two groups. Forest site and city site. Measures included: malondialdehyde (MDA) concentrations, cytokine production, serum cortisol, testosterone assay, lymphocyte assay, POMS evaluations. PP	Data supported the hypothesis that several physiological and psychological metrics were presented in accordance with a decrease in overall stress and subsequent toxic physiologic effects of stress.
Ochiai [25]	Japan	Participants included those recruited from the Health Promotion Center in Agematsu, Nagano Prefecture. All participants needed to be Inclusion aged 40 years or older and deemed healthy at the time of the study.	N = 17 Female adults with an average age of 62.2 ± 9.4 years.	Forest therapy phase was conducted in Akasawa Shizen Kyuyourin, Akasawa Natural Recreation Forest, Agematsu, Nagano Prefecture. Additional assessment took place at the nearby health promotion center.	To assess the psychological and physiological effects of a forest therapy program on middle age adult women. Measures included pulse rate, salivary cortisol levels and psychological indices were taken the day before and the day of forest therapy. PP	Pulse rate, salivary cortisol levels were significantly lower than baseline indicating a physiological relaxed state. Reported significantly more comfortable, relaxed and natural according to the semantic differential. POMS negative mood subscale for tension and anxiety was significantly lower while the “vigor” was significantly higher following forest therapy. A significant decrease in pulse, decrease in salivary cortisol levels, increase in positive feelings, decrease in negative feelings. Substantial benefit to middle age females.
Park [26]	Japan	Participants included young male Japanese university students.	N = 168 Male (100%), mean age 20.4 ± 4.1 years	14 forests and 14 urban areas across Japan	To investigate the relationships between psychological responses and either an urban or a forest setting. Using both the SD method and POMS questionnaire, comparisons were made for both the walking and viewing phases within each area of accommodation. PP	Researchers found that descriptions of the forest area using the SD method were more “enjoyable, friendly, natural, and sacred”. There were also significant differences among POMS results for both the city and forest areas.
Park [27]	Japan	Male University students recruited from Chiba University, Japan.	N = 12; Males with an average age of 22.8 ± 1.4 years.	The experimental trials took place in a Seiwa Prefectural Forest Park in Chiba Prefecture, Japan.	To investigate the physiological effects of Shinrin-yoku using salivary cortisol and cerebral activity as indicators. On the first day of the experiment, one group of 6 subjects was sent to a forest area, the other 6 were sent to a city area. On the second day, each group was sent to the opposite area for a cross check. In the morning, the subjects were asked to walk around their location for 20 min. In the afternoon, they were asked to sit on chairs and watch the landscapes of their set locale for 20 min. Prefrontal cortical cerebral activity and salivary cortisol were measured before and after walking in the forest, or city locations and before and after watching the landscapes in the afternoon in the forest and city areas. PP	Results indicated that cerebral activity in the prefrontal area of the forest area group was significantly lower than that of the group in the city area after walking; the concentration of salivary cortisol in the forest area group was significantly lower than that of the group in the city area before and after watching each landscape. The results of the physiological measurements show that Shinrin-yoku can effectively relax both people’s body and spirit.
Joung [28]	Korea	Eight Korean university students participated in this study. The subjects were deemed physically and mentally healthy prior to the initiation of this study.	N = 8; Participants had an age range of 22.0 ± 2.2 years. Gender undocumented.	Forested-region located in Dowon-ri, Toseong-myun, Goseong-gun, Gangwon-do, Korea. The contrasting urban area was in Yuseong-gu, Daejeon Metropolitan City, Korea.	Determine if forest environments have physiological and psychological relaxing effects by viewing a forest area compared with viewing an urban area from the roof of an urban building without being watched by others. Near-infrared spectroscopy measurement was performed on subjects while they viewed scenery for 15 min. At each experimental site (forest and urban) Total hgb and oxyhemoglobin concentrations were measured. SM	Total hgb and oxyhemoglobin concentrations were significantly lower in their forest area than the urban area. Comfortable, natural, and soothed were significantly higher in the forest vs. urban area. For mood states, the forest group had significantly lower negative emotions.
Mao [29]	China	Subjects included patients diagnosed with essential hypertension in stable condition at the time of the study. All were being treated in Hangzhou, China.	N = 24; Adults, aged from 60 to 75 years, specific demographics unknown.	Broad-leaved evergreen forest “White Horse Mountain National Forest Park” in Suichang, County, Zhejiang Province, China. For comparison, the control city was an urban area in Hangzhou, China.	Quantitative randomized controlled trial was to provide scientific evidence to support the use and efficacy of SY as a practical application for treating, or ameliorating essential hypertension in the elderly. Patients with essential hypertension were randomly divided into a field study group and a control group of 12 persons each. The intervention (field study) group went to a broad-leaved evergreen forest to experience a 7-day/7-night trip, and the control group experienced a city area in Hangzhou for control. Measurements of the following were collected: blood pressure indicators, cardiovascular disease-related pathological factors including endothelin-1, homocysteine, renin, angiotensinogen, angiotensin II, angiotensin II type 1 receptor, angiotensin II type 2 receptor, inflammatory cytokines interleukin-6 and tumor necrosis factor were detected. The profile of mood states (POMS) was used for psychological indicators. PP	Results of this study demonstrated that there is direct evidence to support the application of SY for the amelioration of essential hypertension in the population studied. Data indicates that SY practices contribute to the inhibition of the renin–angiotensin system and inflammation, thereby reducing cardiac workload and further stress on the heart when compared with the urban control.
McCaffrey [30]	USA	Participants included older adults over the age of 65 with depression.	N = 40 (mean age = 71.3 years) with depression diagnosed by a physician.	Morikami Gardens, Florida	To determine the effects of garden walking on depression in older adults. Participants were asked to complete 12 two- hour garden walks during a 3-month period. Throughout the walks, they were asked to read a descriptive paragraph and journal upon reaching specified locations within the gardens. Pictures of these locations were also provided so that journaling could continue when the participants were away from the gardens as well. PP	Mean scores on the Geriatric Depression Scale decreased from 13 to 9.4 after completion of the 12 forest walks.
Kim [31]	Korea	Patients recruited for this study were among a population diagnosed with major depressive disorder at one university hospital located in Seoul, Republic of Korea.	N = 63 males and females; 23 in the forest group, 19 in the hospital group, and 21 in the control group.	Settings were the following; the forest program took place at the Hong-Reung arboretum, while the hospital program took place at the Seoul Paik Hospital.	To test the effect of cognitive behavior therapy (CBT)-based psychotherapy applied in a forest environment on major depressive disorder. Tests used included the Hamilton Rating Scales for Depression (HRSD) scores of the forest group were significantly decreased after 4 sessions compared with controls. Montgomery-Asberg Depression Rating Scales (MADRS) scores of the forest group were significantly decreased compared with both the hospital group and the controls. The remission rate (7 and below in HRSD) of the forest group was 61% (14/23), significantly higher than both the hospital group (21%, 4/19) and the controls (5%, 1/21). PP	CBT-based psychotherapy applied in the forest environment was helpful in the achievement of depression remission, and its effect was superior to that of psychotherapy performed in the hospital and the usual outpatient management.
Morita [32]	Japan	71 healthy adult volunteers participated in this study. Ages ranged from teens to late 70s.	N = 71; 43 males and 28 females.	Ryukoku Forest of Ryukoku University in Shiga Prefecture, located in the western region of Honshu, Japan. Data analysis took place at the University of Shiga, Japan.	Pre and posttest study was to evaluate the immediate effects of forest walking in a community-based population with sleep complaints. Two-hour forest-walking sessions were conducted on 8 different weekend days. Sleep conditions were compared between the nights before and after walking in a forest by self-administered questionnaire and actigraphy data. PP	Results indicated that 2 h of forest walking improved sleep characteristics; impacting actual sleep time, immobile minutes, self-rated depth of sleep, and sleep quality.
Nakau [33]	Japan	Patients were recruited from a pool of cancer patients, specifically those with breast cancer, or lung cancer of various stages. For all participants, one month passed after they had undergone surgery, chemotherapy, or radiation and were in stable condition at the time of study. All participants resided in urban areas and lacked access to green, outdoor environments.	N = 22; Men and women with a mean age of 58.1 years +/10.8 years. Participants included 4 males with an average age of 65.3 and 18 females with an average age of 56.6 years.	Within the Kyoto prefecture of Japan, sites included: The Japan World Exposition 70 Commemorative Park (Suita, Osaka Pref, Japan), parks, forests, and gardens within the park, horticultural settings, participants’ homes, and a local day treatment facility. While watching a yoga video.	To explore the impacts of spiritual care and integration of the natural environments in terms of its’ impact on 22 cancer patients. Specifically, the integrative treatment protocol consisted of forest therapy, horticultural therapy, yoga meditation, and support group therapy sessions were conducted once a week for 12 weeks. The spirituality (the Functional Assessment of Chronic Illness Therapy-Spiritual well-being), quality of life (Short Form-36 Health Survey Questionnaire), fatigue (Cancer Fatigue Scale), psychological state (Profile of Mood States, short form, and State-Trait Anxiety Inventory) and natural killer cell activity were metrics assessed before and after the sessions. PP	There were dramatic shifts pre and post intervention to support the hypothesis aforementioned. Emotional and spiritual health improved for all participants. This study helps to delineate what is meant by “spiritual well-being” with specific questionnaires from which we can glean much in terms of semantics.
Ohtsuka [34]	Japan	Researchers culled their participants from a sample of patients being treated for Type II Diabetes with an age range of 60–83, mean height 154.0 cm ± 1.3, and mean body mass index (BMI) of 23.6 ± 0.4 kg/m^2^. Additionally, researchers incorporated data from longitudinal studies addressing Type II Diabetic patients over a period of 6 years. This increased the sample to 116 persons, from which 25 paired samples were studied. Healthy subjects were used as a control.	N = 48 (16 males and 32 female) Type 2 Diabetic patients with a mean age of 66.8 years.	Research facility and nearby recreational areas in connection with Hokkaido University, Japan.	Quantitative longitudinal study aimed to address the effects of Shinrin-Yoku on blood glucose levels in patients with Type II Diabetes. In an effort to summarize data from future studies, the author of this article noted that an additional sample of 116 persons, organized into 25 paired groups were incorporated. Available data reflects these additional participants. Of the initial sample (N = 48), 11 participants experienced only dietary and exercise therapy, 27 were given oral medication, and 10 were being treated with insulin administration at the time of, and during the study. Pre and posttest measures of blood glucose were taken at specific timed intervals during the intervention process. Participants were assessed after morning meals at the research hospital. Peripheral venous blood samples were collected for glucose levels. Participants were divided into two forest-walking groups. Glucose samples were drawn again post Shinrin-yoku treatment. PP	Results demonstrated that Shinrin-yoku and a decrease in blood glucose are significantly correlated. However, due to the additional longitudinal participant sample being reported in the data, the specificities of the total population are unclear.
Shin [35]	Korea	Subjects were adult males and females diagnosed with alcoholism and coming from treatment at the Korean Alcohol Research Center, Chungbuk Province, South Korea. The Korean Alcohol Research Center is a national inpatient alcohol rehabilitation facility.	N = 92; Adults 84 males, 8 females, aged ~44–49 yeas.	Saneum Recreational Forest, in Kyungggi Province, South Korea.	Quantitative case-control/cohort study with pretest vs. posttest assessments. Subjects were assessed over 9-day while in a forest healing camp in Saneum Recreational Forest, in Kyungggi Province, South Korea, for the determining this therapy’s potential treatment of depression for alcoholics. Measures included The Beck Depression Inventory (BDI), and a self-reported survey of 21 items relating to personal variables and lifestyle metrics. PP	Alcoholics with higher pre-test depression levels improved on the BDI post-test scores upon completion of the forest program more than participants with lower pre-test depression levels. Education level and marital status of participants did not significantly influence results.
Stigsdotter [36]	Denmark	Initial sampling of data from 21,832 adults from Denmark was used for this study. The sample came from a 2005 nationally administered health interview survey employing region-stratified random sampling from the Danish Civil Registration System. 10,250 individuals responded and their data recorded for this study.	N = 10,250; Adult Danes aged 16–75 years, 5802 men and 5448 women.	Utilized data from a previous study taking place across various regions of Denmark by The Danish National Institute of Public Health, University of Southern Denmark.	Case study of pre-existing data. The aim of this study was to research and determine the relevant associations between access to green-space, health, health-related quality of life indicators, and stress. Data was collected from respondents following up of a 2005 Danish Health Interview Survey. The data was collected from face-to-face interviews and self-administered questionnaires. Measures analyzed included: the SF-36, (measuring eight dimensions of health) and the Perceived Stress Scale. Multiple logistic regression analyses were used to determine the association between distance to green space and self-perceptions stress. PP	Results of this study demonstrate that Danish individuals living more than 1 km from green-space reported lower satisfaction of perceived health and quality of life than those living less than 1 km to accessible green-space. Additionally, persons living less than 1 km from a green-space experienced less stress than respondents living farther from green-space. Respondents who didn’t report stress were reported to be more likely to visit green-spaces than respondents reporting stress. Overall, there was a viable association between distance to green-spaces and the health- oriented variables in the research question.
Park [37]	Japan	Male students from the Chiba University, Japan.	N = 12; Male (100%), mean age 21.8 ± 0.8	Conifer forest in Hinokage Town , and Hyuga City in Miyazaki Prefecture, Japan	Quantitative randomized controlled trial forest recreation and its effects on the autonomic nervous system were assessed. By random assignment, two groups were formed into forest-area and urban-area groups. Measures included heart rate and heart-rate variability. The R-R interval of the electrocardiogram was used to analyze how aspects of HRV reflect the parasympathetic nervous activity sympathetic nervous activity respectively. Pulse rate a blood pressure were also measured. PP	Pulse rate, diastolic blood pressure and LF/(LF + HF) (LF—low frequency, HF—high frequency) components of HRV were significantly lower in the forest area than in the city area. HF components of HRV tended to be higher in the forest than in the city. Forest recreation is effective for relaxation of both the mind and body.
Park [38]	Japan	Participants included young male Japanese university students.	N = 12; Male (100%), mean age 21.3 ± 1.1	Areas of study included Shinano town and Nagano city in Nagano Prefecture.	To determine the physiological effects of SY. Day one of the experiment required that six subjects went to the forest area, and the other six went to a city area. On the second day, subjects went to the opposite of their previously assigned areas. During the morning and evening within the area of accommodation, heart rate variability (HRV), salivary cortisol and pulse rate were measured, In the afternoon, they were seated on chairs watching the landscapes of their given area for 15 min. The aforementioned physiological indices were again measured before and after watching the landscapes in the given field areas. PP	Researchers found that HRV of subjects in the forest area was significantly higher than that of subjects in the city area. On the other hand, both pulse rate and salivary cortisol concentration of subjects in the forest area was significantly lower than that of subjects in the city area.
Song [39]	Japan	Male students from the Chiba University, Japan.	N = 23; Male (100%), mean age 22.3 ± 1.2	Kashiwa-no-ha Park in Kashiwa City, Chiba Prefecture, Japan, with a nearby city area denoted as the urban control site.	Quantitative. Non-randomized controlled trial, within-subjects design. The aim of this study was to demonstrate how the intervention of walking in urban parks during the fall season impacted participants’ heart-rate and stress levels. Students walked 15 min each on specific trails in a park and in a nearby urban area (the control). HR, HRV, the State Trait Anxiety Inventory, and POMS, were measured to assess the difference outcomes between walk-sites. PP	The walk in the park enhanced relaxation in the participants via parasympathetic nervous system stimulation, while sympathetic nervous system stimulation was decreased. Heart-rate lowered overall. Suggests the effectiveness of even “small” green areas on heart-rate variability.
Tsunetsugu [40]	Japan	Male university students were recruited for this study. All were deemed healthy at the time of the trial.	N = 12; Males aged 21 to 23 (mean ± SD: 22.0 ± 1.0).	Conducted in a broadleaf forest mainly Nukumidaira, Oguni, Yamagata, Japan.	To study the physiological effects of SY were examined by investigating blood pressure, pulse rate, heart rate variability (HRV), salivary cortisol concentration, and immunoglobulin A concentration in saliva. Subjective feelings of being “comfortable”, “calm”, and “refreshed” were also assessed by questionnaire. Physiological measurements were conducted six times, i.e., in the morning and evening before meals at the place of accommodation, before and after the subjects walked a predetermined course in the forest and city areas for 15 min, and before and after they sat still on a chair watching the scenery in the respective areas for 15 min. PP	Data of the study revealed that blood pressure and pulse rate were significantly lower, and that the power of the HF component of the HRV tended to be higher and LF/(LF + HF) tended to be lower. Salivary cortisol concentration was significantly lower in the forest area, and feelings of comfort were significantly higher in the forest area.
Song [41]	Japan	Participants recruited for this study were adult male Japanese citizens with a history of prehypertension and/or current hypertension deemed in suitable physical condition to participate in this study.	N = 20; Adult men with a mean age of 58.0 ± 10.6 years.	Akasawa Shizen Kyuyourin; Akasawa natural recreation forest within Agematsu town of Nagano Prefecture in central Japan. The control was a city area within A City of Nagano Prefecture, Japan.	To look at the effects of forest walking on the autonomic nervous system in middle aged hypertensive adults. Subjects were instructed to walk predetermined courses in forest and urban (control). The course length was 17-min. Walk walking speed and energy expenditure were equal between both groups. HRV and HR were used to quantify physiological responses. PP	HR significantly lower and high frequency component of HRV was significantly higher. Questionnaire results indicate after walking in the forest the feelings were increased around comfortable, relaxed, natural, vigorous, decreased tension and anxiety, depression, anxiety hostility, fatigue and confusion. A brief walk in the forest elicited psychological relaxation and physiological calm on the subjects.
Kardan [42]	Canada	Large urban population in Toronto, Canada.	Tree lined streets in urban neighborhoods.	The study was conducted in Toronto, Canada.	Multivariate study combining high-resolution satellite imagery and individual tree data from Toronto with self-reports of general health perception, cardio-metabolic conditions and mental illness derived from the Ontario Health Study.	Having 10 or more trees in a city block improves health perception in a way that is like an increase in annual personal salary of $10,000. And, having 11 more trees in a city block decreased cardio-metabolic conditions in ways compared to an increase in an annual personal income of $20,000.
Grazuleviciene [43]	Lithuania	20 male and female residents of Kaunas, Lithuania each with a diagnosis of Coronary Artery Disease and cardiac comorbidities being treated at the Cardiologic Clinic of the Hospital of Lithuanian University of Health Sciences.	N = 20; Male and female participants with a mean age of 62.3 ± 12.6 years.	The study was conducted in Kaunas. The urban exposure area was a street near the Hospital of Lithuanian U. Cardiology Clinic. The green exposure region was a pine tree park located near the Cardiology Clinic.	Quantitative Randomized Controlled Trial was to study the impact of forest-walking on patients being treated for CAD. Participants were randomly assigned to either green or urban exposure groups and walked in these different environments for 30 min on 7 consecutive days. Researchers aimed to determine how the different environments impacted patients’ hemodynamics and state of their CAD diagnoses. Testing involved pretest phenotype questionnaires, various health assessment tools including: SBP, DBP, HR, PWV, ECG, W (workload), Spiroergometry. PP	Walking in a park had a more positive effect on overall cardiac function in patients than walking in urban environments.
Jia [44]	China	Adult patients diagnosed with Chronic Obstructive Pulmonary Disease, from the region of Hangzhou, China, with no exacerbations of COPD within 6 weeks of the trial.	N = 20; male and female adult participants aged 60 to 79 years.	Hangzhou, China	Elucidate health effects of a FB trip on elderly patients with chronic obstructive pulmonary disease (COPD). Subjects were randomly divided into two groups. One group was sent to forest, and the other was sent to an urban area as control. Flow cytometry, ELISA, and profile of mood states (POMS) were evaluated. PP	Within the forest group, there was a significant decrease of perforin and granzyme B expressions, accompanied by decreased levels of pro-inflammatory cytokines and stress hormones. Meanwhile, the scores in the negative subscales of POMS decreased after FB trip. These results indicate that FB trip has health effect on elderly COPD patients by reducing inflammation and stress level.
Ohtsuka [45]	Japan	Researchers culled their participants from a sample of patients being treated for Type II Diabetes with an age range of 60–83, mean height 154.0 ± 1.3 cm, and mean body mass index (BMI) of 23.6 ± 0.4 kg/m^2^. Additionally, researchers incorporated data from longitudinal studies addressing Type II Diabetic patients over a period of 6 years. This increased the sample to 116 persons, from which 25 paired samples were studied. Healthy subjects were used as a control.	N = 48 (16 males and 32 female) Type 2 Diabetic patients with a mean age of 66.8 years.	Research facility and nearby recreational areas in connection with Hokkaido University, Japan.	Quantitative longitudinal study aimed to address the effects of Shinrin-Yoku on blood glucose levels in patients with Type II Diabetes. In an effort to summarize data from future studies, the author of this article noted that an additional sample of 116 persons, organized into 25 paired groups were incorporated. Available data reflects these additional participants. Of the initial sample (n = 48), 11 participants experienced only dietary and exercise therapy, 27 were given oral medication, and 10 were being treated with insulin administration at the time of, and during the study. Pre and posttest measures of blood glucose were taken at specific timed intervals during the intervention process. Participants were assessed after morning meals at the research hospital. Peripheral venous blood samples were collected for glucose levels. Participants were divided into two forest-walking groups. Glucose samples were drawn again post Shinrin-yoku treatment. PP	NA
Morita [46]	Japan	71 healthy adult volunteers participated in this study. Ages ranged from teens to late 70s.	N = 71; 43 males and 28 females.	Ryukoku Forest of Ryukoku University in Shiga Prefecture, located in the western region of Honshu, Japan. Data analysis took place at the University of Shiga, Japan.	Pre and posttest study was to evaluate the immediate effects of forest walking in a community-based population with sleep complaints. Two-hour forest-walking sessions were conducted on 8 different weekend days. Sleep conditions were compared between the nights before and after walking in a forest by self-administered questionnaire and actigraphy data. PP	Results indicated that 2 h of forest walking improved sleep characteristics; impacting actual sleep time, immobile minutes, self-rated depth of sleep, and sleep quality.
Sung [47]	The Republic of Korea	Recruitment included stable patients with stage 1 HTN, and/or patients who were on antihypertensive medication.	N = 56; Males and females aged 63–73 years.	The forest group participated at two recreation forest sites including Hoengseong and Saneum, in Kangwon-do, Republic of Korea. The control group maintained regular treatment at the treatment facility.	To study the effects of a Forest Therapy/CBT-based community program on adult patients with HTN, referred from two local health centers in Seoul, S. Korea. This study was a controlled trial without true randomization. For an 8-week intervention duration of the treatment protocol. Data included a comparison of pre and post intervention measures of: BP, A. Qol. (Quality of Life survey tool), and salivary cortisol measurements of control group vs. Forest Therapy program group, SBP and DBP manual measurements. PP	Forest Therapy/CBT-based community program may have initially reduced SBP measures, marked decrease in salivary cortisol levels, and improvement in A Qol measures.
Largo-Wight [48]	USA	Full-time, desk-bound, and otherwise sedentary office staff (secretaries and clerks), at an undisclosed southeastern university in Florida, USA.	N = 503. Office staff at a southeastern university. Response rate (30%). Women (92.9%) Caucasian (82.5%). Mean age (42 years; SD 12 years) Appx. (47.5%) of all participants attended college or technical school. (49.5%) reported annual income of $25,001–$35,000 per year. (54.4%) reported being married.	Workplace (office) environment at a southeastern university.	Quantitative study with a cross-sectional design. Employed all web-based questionnaires including (via email invitation); 16-item survey on workplace environs, the Nature Contact Questionnaire (NCQ), The Perceived Stress Questionnaire (PSQ) and 13-item health behavior assessed dependent variables and health outcomes. web-based survey design. PP	Significant, negative association between nature contact and stress and nature contact and general health complaints. The results indicate that as workday nature contact increased, perceived stress and generalized health complaints decreased.
Takayama [49]	Japan	Participants were recruited from four prefectures in Japan. All participants were male university students deemed healthy at the time of the study.	N = 45; Adult males aged 19–22 years.	Forested and urban sites (8 total)), were used in this study. All sites located in, or near to the towns of Yoshino, Akiota and Kamiichi and the city of Oita, Japan.	To test the beneficial health effects of walking in forests against urban environs in 45 total respondents. Four nature walking sites and an urban control were used as the field sites. This quantitative study included the following psychological assessment tools: Profile of Mood States (POMS) indexes, Restorative Outcome Scale (ROS) and Subjective Vitality Scale (SVS). PP	Hypothesis was supported—in that- compared with the urban control, overall psychological well-being improved more in forest environments. Subjects noted that the forest walking program induced feelings of relief and revitalization, whereas the urban walks did not.
Kang [50]	Korea	Participants for this study were recruited from the Department of Rehabilitation Medicine of Hanyang University Medical Center, where they were being treated with posterior neck pain for a period of 3 months or greater.	N = 64; 11 males and 53 females.	Experimental trials took place at the Department of Rehabilitation Medicine of Hanyang University Medical Center and undisclosed nearby forested area.	To compare the pain-reducing effect of FB alone vs. FB in combination with stretching and strengthening exercises in patients with chronic posterior neck pain. Participants were randomly divided into FB Alone (FBA: n = 32) and FB Exercise (FBE: n = 32). The Visual Analog Scale, neck disability index (NDI), Euro-Qual 5D-3L VAS (EQ VAS) and index (EQ index). McGill pain questionnaire (MPQ), number of trigger points in posterior neck region (TRPs) and ROM of cervical spine were evaluated on the first and last day of the program and compared between groups. PP	The number of TRPs were significantly reduced in the FBE group compared with the FBA group (*p* = 0.013). The other scales used showed no difference.
Hawker [51]		Visual Analog Scale reliability and validity	NA	NA	Measures of adult pain: Visual Analog Scale for Pain (VAS Pain), Numeric Rating Scale for Pain (NRS Pain), McGill Pain Questionnaire (MPQ), Short-Form McGill Pain Questionnaire (SF-MPQ), Chronic Pain Grade Scale (CPGS), Short Form-36 Bodily Pain Scale (SF-36 BPS), and Measure of Intermittent and Constant Osteoarthritis Pain (ICOAP)	NA
Beil [52]	USA	Participants included residents of Portland, Oregon.	N = 15; 15 (8 males, 7 female), aged 20–61 years with an average age of 42.3.	Urban and natural outdoor settings (4 total) within 15 km of research lab.	Quantitative, randomized controlled trial with pre- and post-test design. Participants were exposed to urban and natural forested environmental settings respectively for 20 min at a time. Salivary amylase and subjective measures of stress were taken before and after each exposure. Testing methods included web-based survey analyses via the Subjective Stress Scale (Stress), Environmental Identity (EID) Scale Perceived Stress Scale (PSS) Perceived Restrictiveness Scale (PRS) [post-test only], Saliva (sCort and sAA). PP	Participants experienced less physiological & psychological stress from exposure to the natural environments versus built environs as measured by pre- and post-intervention changes in salivary amylase and self-reported stress. The greatest decrease in stress was noted in females in natural settings.
Kobayashi [53]	Japan	The study consisted of 625 male Japanese students at the University of Chiba, Japan, deemed healthy at the time of the study.	N = 625; Males with a mean age of 21.6 years +/1.6 years.	Forested areas (57), and urban areas (57) within Japan.	To expose Japanese students at the Chiba University to urban and forest environments respectively—in order to ascertain relevant effects on autonomic nervous system function. This quantitative RCT included 57 Forest and 57 Urban sites selected across Japan. Participants sat for 15 min while viewing either setting. Measures included HRV, which was monitored continuously. The experiment was performed over 2 consecutive days at each site. Measures of HRV were conducted between 13:30 and 15:30 for 15 min at a time. These were RCTs; one group was exposed to the forest site prior to the urban site & vice versa. PP	Demonstrated a roughly 80% increase in the parasympathetic indicators of HRV with a decrease in sympathetic indicators of HRV—physiologically demonstrating that forest-viewing was more effective in reducing indicators of stress than the urban areas.
Engert [54]		Supporting material	NA	NA	Investigation into the cross-correlation of salivary cortisol and alpha-amylase responses to psychological stress.	NA
Fisher [55]		Supporting material	NA	NA	A Conversation with David Milarch	NA
Igarashi [56]	Japan	Students (male and female), from the Chiba Prefectural Kashiwanoha Senior School, deemed in good health prior to the orientation of the study.	N = 48; 19 high school males mean age 16.2 ± 0.7 years, and 21 high school females mean age 16.6 ± 0.9 years.	Chiba Prefectural Kashiwanoha Senior High School lab rooms.	Clarification on the physiological and psychological effects on HS students viewing real vs. artificial pansies. Participants were exposed to yellow fresh pansies for 3 min each. Artificial pansies in a planter were used as a control. Heart rate variability (HRV) was tested. SM	Exposure to real pansies increased the activity of the Parasympathetic Nervous System (PNS). Viewing real flowers resulted in comfortable relaxed and natural feelings. Visual stimulation with real flowers induced psychological relaxation effects HS students
Tsutsumi [57]	Japan	Participants included young male Japanese adults deemed healthy at the time of study.	N = 12; Males, with a mean age of 22.2 ± 1.7 years.	Studies completed in laboratory settings at the research center.	To determine whether stimulation by viewing an individual preferred video of sea or forest has an effect on relaxation. Participants were divided into two groups based on their preference for sea or forest scenery. By using a visual analog scale the participants watched 90 min. DVDs of the sea with natural sounds and forest with natural sounds while HR variability and Bispectral Index System value were measured using MemCalc/Tawara and Bispectral Index System monitor. SM	Decreased HR, increase in high frequency and sustained arousal level were observed while viewing the preferred video. The viewing of the preferred video had a positive relaxation effect. Individual preferences should be honored when initiating video relaxation therapy.
Park [58]		Supporting material	NA	NA	The physiological effects of Shinrin-yoku (taking in the forest atmosphere or forest bathing): Evidence from field experiments in 24 forests across Japan.	NA
Logan [59]	USA	Supporting material	NA	NA	Literature review is based upon 30 years of available research demonstrating the potential healing properties of nature on mental and physical well-being under the premise of nature’s healing power coined by Sir. J. Arthur Thomson. The authors propose a philosophical and psychological framework from which to conceive of the potential for forest-therapy and forest-bathing programs F	Research culled demonstrated that “nature” exposure is beneficial to the mind in terms of relaxation and feelings of connectedness - as are viewed by the tenets of this paper to be “beneficial.”
Selhub [60]		Supporting material	NA	NA	The Science of Nature’s Influence on Your Health	NA
Berger [61]	USA	Supporting material	NA	NA	Describes a creative framework in which nature is incorporated into therapy with older adults. Using a practical example, this study illustrated how the integration of concepts from a narrative approach and the innovative nature-therapy framework could aid a geriatric population in expanding personal perspectives, strengths, and coping strategies, while gaining a wider sense of acceptance and completion in life. F	This framework highlighted a way in which the connection between the personal story and the natural, cosmic story could enhance the participants’ sense of completion within themselves and their surroundings, which may be further implemented into holistic nursing.
Zdravkovic [62]		Supporting material	NA	NA	Nature and forest therapy workshops offered online.	NA
Poulsen [63]	Denmark	The population addressed were soldiers and veterans from the Danish military rehabilitation unit who had served in war and were diagnosed with some degree of Post-Traumatic Stress Disorder.	N = 8; Males aged 26–47 years.	University of Copenhagen forest therapy garden “Nacadia,” which is located in Hørsholm arboretum.	Qualitative study employed a phenomenological approach in an effort to understand and elucidate the effects of Nature-Based Therapy (NBT), on the well-being of the subjects. The design of the study was based on a previous case study from China addressing similar questions. In this study the participants PTSD is the concern, the context is the forest therapy garden and the phenomenon is the participant’s experience of NBT from 10 consecutive weeks of treatment. Interviews were conducted at four stages of the program. PP	Researchers generated three key themes from analysis of the interviews. These included: taking nature in, Nature-Based association as an initiation to therapeutic processes, and nature seen as a part of everyday life. Participants reported a sense of refuge, safety, calmness, and general wellbeing.
Beck [64]		Supporting material	NA	NA	Perceived level and sources of stress in baccalaureate nursing students.	NA
Reeve [65]		Supporting material	NA	NA	Perceived stress and social support in undergraduate nursing students’ educational experiences.	NA
Kreitzer [66]		Supporting material	NA	NA	Integrative nursing: Application of principles across clinical settings.	NA
Watson [67]		Supporting material	NA	NA	The core concepts of Jean Watson’s Theory of Human Caring and Caring Science.	NA
Olsson [68]	Sweden	Population criteria included males and females from a region in Sweden diagnosed with early-stage dementia of various ages, who were able to speak. Sampling included persons who had an expressed desire to be alone outdoors and were living at home.	N = 11; Aged between 52 to 81 years, 5 women and 6 men.	Interviews in this study took place in the participant’s homes; indoors and outdoors at the homes.	Qualitative interview based study was to determine and report on how persons diagnosed with dementia reflect on what it’s like for them when experience an outdoor setting. Purposive sampling was used to obtain this cohort. As part of a larger study focusing on dementia, qualitative content analysis was used to categorize the interview results into core themes based upon interviewee’s experiences and discussion. PP	Results of these interviews reflected that subjects unanimously experiences a sense of well-being and self-worth regarding independent outdoor activity. Furthermore, interviewees reported the outdoor setting as complementary to a sense of well-being. Potential challenges and adaptive strategies were addressed when navigating outdoor settings.
Song [69]	Japan	Middle-age hypertensive men, that had never taken medication for HTN, HLD, or DM	N = 20; Male (100%, )mean age 58.0 ± 10.6 years	Akasawa natural recreation forest near the Agematsu town of Nagano Prefecture	To determine the effects of FB on the autonomic nervous system. Participants were asked to sit in both urban and forest areas for 10 min in each location and Heart Rate were both measured. Questionnaires were also given to participants to collect data on emotional condition while viewing both settings. PP	HF HRV was increased while viewing a forest landscape, while heart rate was decreased in comparison to the urban setting viewings. Additionally, participants felt more “comfortable,” “relaxed,” and “natural” after viewing the forest.
Berger [70]	USA	Supporting material	N/A	N/A	Proposed an application of NT for the treatment of emotional and psychiatric issues. This expository piece provided a framework based upon previous applications of NT in two anecdotal examples from Israel: “The Healing Sand” and “The Enchanting Forest.” F	The innovative approach offered in this article was supported by background research in relevant expressive arts therapies. It inferred that NT enables persons to feel relief from stress in addition to increased social conscientiousness.
Berger [71]	Israel	The population at the center of this study included students in the elementary classroom of Galim as well as their homeroom teacher and therapist.	N = 13; 9 boys and 2 girls aged 7–9, 2 female adults aged 42 and 33, respectively.	Galim elementary school (Israel) for children with learning and behavioral disabilities.	Expositional case study, rooted in grounded theory analysis, qualitatively analyzed the utilization of the NT framework for children with learning and behavioral disabilities. Subjects engaged in a NT program for 1 year. Interviews and questionnaires provided data based upon analysis of the framework’s role in education and scholastic development. PP	This study demonstrated a successful protocol and application for nature -oriented therapeutic activities in a classroom setting for study population and described how NT can enhance, or support pre-existing modalities for students with learning disabilities.
Berger [72]	USA	Supporting material	N/A	N/A	NT created theories and models to assist the therapist working in nature to create a therapeutically appealing setting. From a psychological, eco-social perspective, the author surmised that strengthening a relationship with nature may reduce depression and anxiety and foster emotional continuity, happiness and wonder. The purpose of this book chapter (#2) was to illustrate a framework for NT as a creative therapeutic discipline via active research and reflexive process for the author’s PhD. F	This framework showcased concepts from eco-psychology, drama and ritual.

Key: PP: Psychological & Physiological. SM: Sense Metrics. F: Frameworks.
